# Rare Cardiac Tumor: Do We Know All About Cardiac Myxofibrosarcoma?

**DOI:** 10.7759/cureus.58000

**Published:** 2024-04-10

**Authors:** Saloni Savani, Arpita Pawa, Het Patel, Mohammed Syed, Samip Master

**Affiliations:** 1 Internal Medicine, Willis-Knighton Health System, Shreveport, USA; 2 Hematology and Oncology, Willis-Knighton Health System, Shreveport, USA

**Keywords:** mitral valve surgery, doxorubicin and ifosfamide, myxofibrosarcoma, embolic stroke, primary cardiac tumor

## Abstract

Primary cardiac tumors (PCTs) are less frequent and carry an incidence of 1.38 per 100,000 population per year. Myxofibrosarcomas are reported as one of the rarest forms of cardiac sarcomas, mostly with mesenchymal origin and located in the left atrium. Current research indicates an increase in median survival from 14 months to 36 months following complete resection and chemoradiotherapy. A 55-year-old Caucasian woman was admitted with brief self-resolving episodes of aphasia following migraine headaches for the past few months with associated exertional dyspnea and episodes of hypotension. Examination revealed a right-sided facial droop with cardiac murmur on auscultation. MRI brain was recommended which revealed a non-hemorrhagic infarct and multiple watershed infarcts. A transesophageal echocardiography revealed a large mass of around 5 cm in size located at the posterior wall of the left atrium causing mitral stenosis. The patient was initially managed conservatively and referred to cardiothoracic surgery and underwent a complete surgical resection. The histopathological report indicated the presence of primary cardiac sarcoma, and a postoperative positron emission therapy (PET) scan revealed no other foci of cancer further strengthening evidence of a primary cardiac pathology. This case represents a rare cardiac pathology presenting with non-cardiac symptoms.

## Introduction

Cardiac tumors are uncommon and can be classified as primary or secondary depending on their origin. Primary cardiac tumors arise from the cardiac tissue, while secondary tumors metastasize to the heart and are more commonly reported. Primary cardiac tumors (PCTs) are rare and carry an incidence of 1.38 per 100,000 population per year with primary cardiac sarcomas accounting for about 20% of all primary cardiac tumors [1,2]. Myxofibrosarcomas are reported as one of the rarest forms of cardiac sarcomas [2]. Most of them are of mesenchymal origin and can be found in the atria, ventricles, and (blood) vessels such as pulmonary veins, pulmonary arteries, and aorta. The diagnosis is usually late and/or when the patient develops obstructive symptoms, thromboembolic events, or metastasis [3,4]. Current research indicates an increase in median survival from 14 months to 36 months following complete resection and chemoradiotherapy [2]. We report a patient with primary cardiac myxofibrosarcoma who presented with stroke, hypotension, and dyspnea.

## Case presentation

A 55-year-old Caucasian female with a past medical history of essential hypertension, migraine headaches, gastroesophageal reflux disease due to eosinophilic esophagitis, and generalized anxiety disorder was admitted to the hospital from her primary care physician’s (PCP) office after magnetic resonance imaging (MRI) brain revealed a focal area of restricted diffusion in the right superior parietal cortex compatible with a non-hemorrhagic infarct and a small old left cerebellar infarct.

Of note, the patient had been experiencing self-limiting episodes of memory loss and aphasia following migraine headaches for the past one month and was undergoing workup with her PCP. An initial computed tomography scan of the head was negative for any acute abnormalities. She was diagnosed with migraine headaches with neurological sequelae and managed appropriately. Her symptoms, however, continued to persist, and she subsequently underwent an MRI brain that revealed a stroke. At the time of admission, she also reported fatigue and dyspnea on exertion but denied chest pain, leg swelling, lightheadedness, dizziness, syncope, fever, chills, weight loss, or any sensory or motor loss. Physical examination revealed a right-sided facial droop and a diastolic murmur near the apex on auscultation. Vitals were significant for hypotension with blood pressure of 88/56 mm Hg, heart rate of 92 beats/min, and respiratory rate of 16 breaths/min. Her social history was significant for occasional alcohol use; however, she denied tobacco or illicit drug abuse. Family history was remarkable for diabetes mellitus, stroke, coronary artery disease, and brain angiomas.

**Figure 1:**
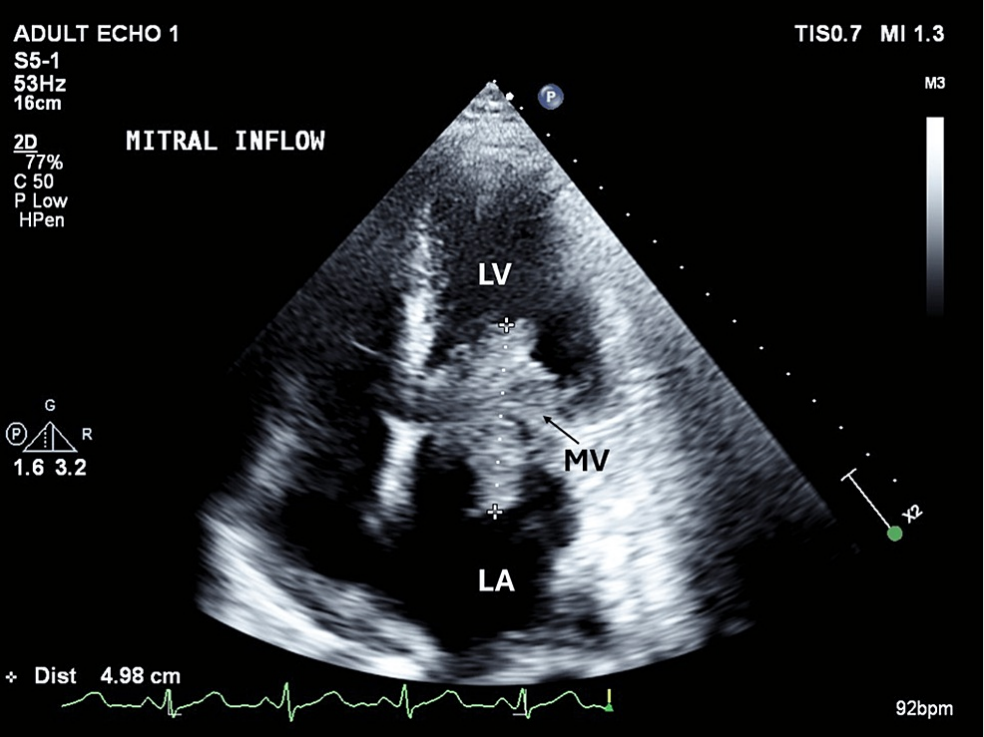
Transesophageal echocardiogram Showing around 5 cm mass attached to the left atrial (LA) wall and posterior leaflet mitral valve (MV) (arrow) with prolapse of segments of the mass into the left ventricle (LV) causing obstruction.

**Figure 2:**
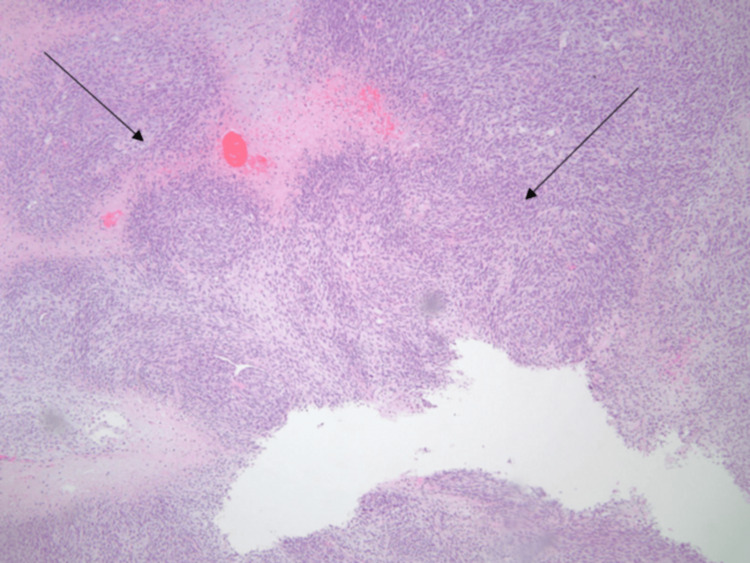
Histological Image of the tumor in 4x magnification. The histopathological image of the mass indicating the proliferation of malignant spindle cells (arrows).

**Figure 3:**
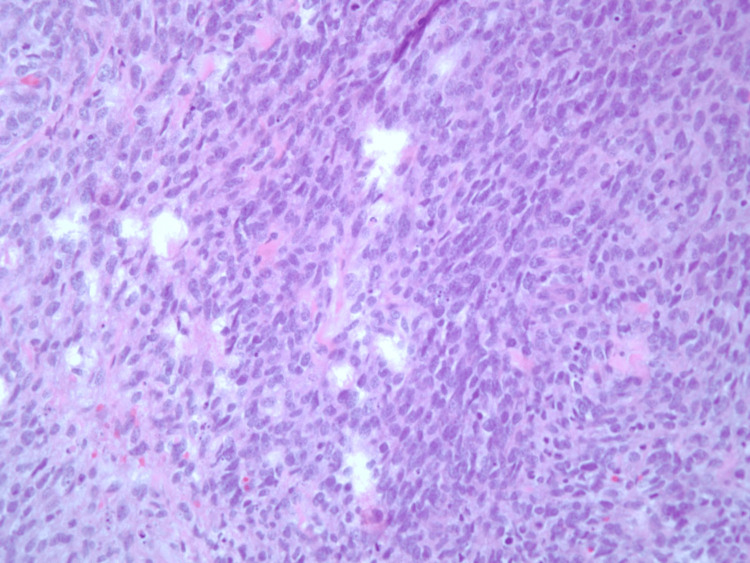
Histological Image of the tumor in 40x magnification The histopathological image of the mass indicating the proliferation of malignant spindle cells.

Upon admission, she underwent a comprehensive stroke workup with routine lab investigations, electrocardiogram (EKG), transthoracic echocardiogram (TTE), and CT angiography of the head and neck. EKG was suggestive of tachycardia with low voltage QRS complexes, while echocardiogram revealed a large 5 cm mass in the left atrium attached to the left atrial wall and posterior leaflet of the mitral valve with partial prolapse of the segments of the mass into the left ventricle causing obstruction (Figure 1). The ejection fraction was >60% with moderate left atrial enlargement and elevated pulmonary artery pressures. The patient was initiated on heparin infusion, and Cardiology and Cardiothoracic surgery were consulted. She subsequently underwent complete excision of the mass with mitral valve repair and had an uneventful postoperative period. The pathology of the specimen indicated the proliferation of malignant spindle cells with foci of necrosis and prominent mitotic figures with Fédération Nationale des Centres de Lutte Contre le Cancer (FNCLCC) grade 3 of 3. The spindle cell population was positive for CD31 and weakly positive for pan-cytokeratin highlighting the presence of a cardiac sarcoma (Figures 2, 3).

**Figure 4:**
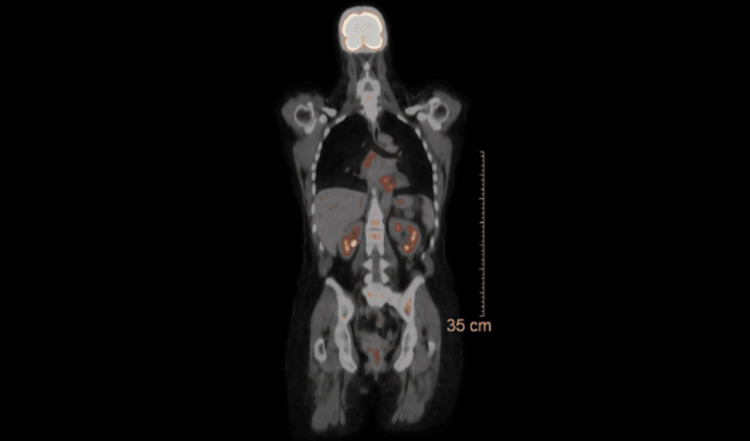
Positron emission therapy scan showing only reactive changes in lower paratracheal, subcarinal, and cervical lymph nodes

Hematology-Oncology was consulted, and the patient underwent a positron emission therapy scan postoperatively that showed only reactive changes in the lower paratracheal, subcarinal, and cervical chain at level 4 and on the right side, further strengthening the evidence of a primary cardiac myxofibrosarcoma (Figure 4). She then underwent a repeat MRI brain for complete staging that revealed metastatic lesions, and whole brain radiation therapy was initiated. Concurrently, she was started on Doxorubicin 75 mg/m^2^ bolus plus Zinecard on day 1 and Ifosfamide 10 mg/m^2^ in a divided dose over five days for a total of five cycles after which her treatment was held owing to poor tolerance.

A month after stopping therapy, restaging scans with cardiac MRI, MRI brain, and chest CT were negative for any recurrence or metastatic disease. It was decided to continue holding treatment with routine monitoring for recurrence every three months.

## Discussion

Through this case, we would like to highlight the severity of the presentation despite minimal symptoms. The patient’s tumor involved the left atrial wall and posterior leaflet of the mitral valve, which itself gives high risk of left ventricular inlet obstruction and thromboembolic events. This patient presented following a stroke, with episodes of hypotension and tachycardia in addition to exertional dyspnea that prompted further diagnostic workup. Considering the rarity of sarcomas and heterogeneity of these malignant tumors of mesenchymal origin that comprise less than 1% of all adult malignancies, early diagnosis and management is the key to better outcomes [5].

Primary tumors of the heart are rare with sarcomas being the rarest. Angiosarcoma is the most common, whereas myxofibrosarcoma and osteosarcoma are the least common of the various primary cardiac sarcomas. As per a report, myxofibrosarcoma comprises 5% of all sarcomas. Clinical presentation ranges from incidental discovery on imaging tests to lethal presentation such as cardiac tamponade, arrhythmia, obstruction, and systemic embolization [6]. TTE is the leading imaging modality for primary detection of a cardiac mass; however, in recent years, cardiac CT and MRI have emerged as routine investigations for diagnosing structural heart diseases [5].

Sun et al. in their case report with literature review and pooled analysis measured the risk factors related to worst outcomes following cardiac myxofibrosarcoma [7]. They came up with the findings of mean survival of around 33 months and suggested that primary cardiac myxofibrosarcomas were more likely to present with local recurrences than distant metastasis. Echocardiography together with histopathological testing should be considered for diagnosis. Tumor size of >4 cm or with high grade was found to be independently associated with a significantly worse prognosis [7]. In this case, the patient had a large 5 cm mass occupying a significant part of the left atrium necessitating complete surgical removal and adjuvant chemotherapy for improved survival. The median age of survival of patients diagnosed with primary cardiac tumors was historically close to one year, but with the advent of multimodality imaging and newer chemotherapeutics, this age has increased to close to three years [2].

## Conclusions

In this instance, the patient's presentation with nonspecific symptoms such as aphasia and exertional dyspnea prompted the initiation of diagnostic investigations. Given the rarity of primary cardiac tumors and the absence of reliable early detection methods, it is imperative to maintain a cautious approach in suspecting these tumors. Preferred investigative modalities include transthoracic echocardiography (TTE), cardiac computed tomography (CT), and magnetic resonance imaging (MRI). A multimodal treatment approach was employed in this case, involving surgical resection, chemotherapy, and radiation therapy, resulting in the patient being tumor-free upon completion of treatment. Historically, the survival rates have been grim, but with the advent of newer diagnostic and treatment modalities, mean survival has improved significantly.
